# Transaxillary implantation of a temporary microaxial left ventricular assist device in a patient with a rectangular kinked subclavian artery

**DOI:** 10.1093/icvts/ivad088

**Published:** 2023-06-05

**Authors:** Leonhard Wert, Jörg Kempfert, Volkmar Falk, Evgenij V Potapov

**Affiliations:** Department of Cardiothoracic and Vascular Surgery, Deutsches Herzzentrum der Charité (DHZC), Berlin, Germany; Department of Cardiothoracic and Vascular Surgery, Deutsches Herzzentrum der Charité (DHZC), Berlin, Germany; DZHK (German Center for Cardiovascular Research), Partner Site, Berlin, Germany; Department of Cardiothoracic and Vascular Surgery, Deutsches Herzzentrum der Charité (DHZC), Berlin, Germany; DZHK (German Center for Cardiovascular Research), Partner Site, Berlin, Germany; Department of Cardiothoracic Surgery, Charité − Universitätsmedizin Berlin, Corporate Member of Freie Universität Berlin, Humboldt-Universität zu Berlin and Berlin Institute of Health, Berlin, Germany; Department of Health Sciences and Technology, ETH Zurich, Zurich, Switzerland; Department of Cardiothoracic and Vascular Surgery, Deutsches Herzzentrum der Charité (DHZC), Berlin, Germany; DZHK (German Center for Cardiovascular Research), Partner Site, Berlin, Germany

**Keywords:** Cardiogenic shock, Impella, Temporary microaxial left ventricular assist device

## Abstract

Transaxillary implantation of a temporary microaxial left ventricular assist device in patients suffering from cardiogenic shock is an established technique. We present a 77-year-old female patient with severe mitral regurgitation. She underwent minimally invasive surgical mitral valve replacement. After an uneventful postoperative course, the patient developed acute heart failure on the 11^th^ postoperative day. Transthoracic echocardiography revealed new onset of Takotsubo cardiomyopathy with a severely decreased left ventricular ejection fraction. Implantation of a microaxial flow pump for left ventricular decompression was scheduled. Preoperative computed tomography revealed a rectangular course of the right subclavian artery. To advance the Impella, we employed an introducer fitted over the guidewire behind the Impella device as a ‘cue stick’ to move the rigid part of the pump forward to overcome the kinking using a ‘shuffleboard technique’. The haemodynamic situation stabilized immediately after implantation. The Impella 5.5 was successfully weaned after 6 days of support. In the event of (rectangular) kinking of the subclavian artery, the ‘shuffleboard technique’ can be used for the successful positioning of the pump.

## INTRODUCTION

Transaxillary implantation of a temporary microaxial left ventricular assist device (Impella 5.5, Abiomed Inc., USA) in patients suffering from cardiogenic shock is an established technique [1]. Here, we report on the first-in-man successful implantation of a microaxial flow pump in a rectangular kinked subclavian artery. The device could be safely deployed in a ‘shuffleboard technique’.

## CASE REPORT

A 77-year-old female patient (165 cm, 75.1 kg, body mass index 27.58 kg/m^2^) presented with primary severe mitral regurgitation and two-vessel coronary artery disease (CAD). She was admitted with dyspnoea (NYHA class III) and recurrent cardiac decompensation. Her history included ischaemic cardiomyopathy, which was first diagnosed in 2019. The left ventricular ejection fraction was 60% in terms of a heart failure preserved ejection fraction. CAD had been treated with percutaneous transluminal coronary angioplasty and drug-eluting stents and currently required no intervention. Preoperative angiography excluded the progression of CAD.

She underwent minimally invasive surgical mitral valve replacement with a 29-mm biological prosthesis in a standard right lateral mini-thoracotomy approach. The postoperative course was uneventful with extubation and transfer to the normal ward on the same day.

**Figure 1:**
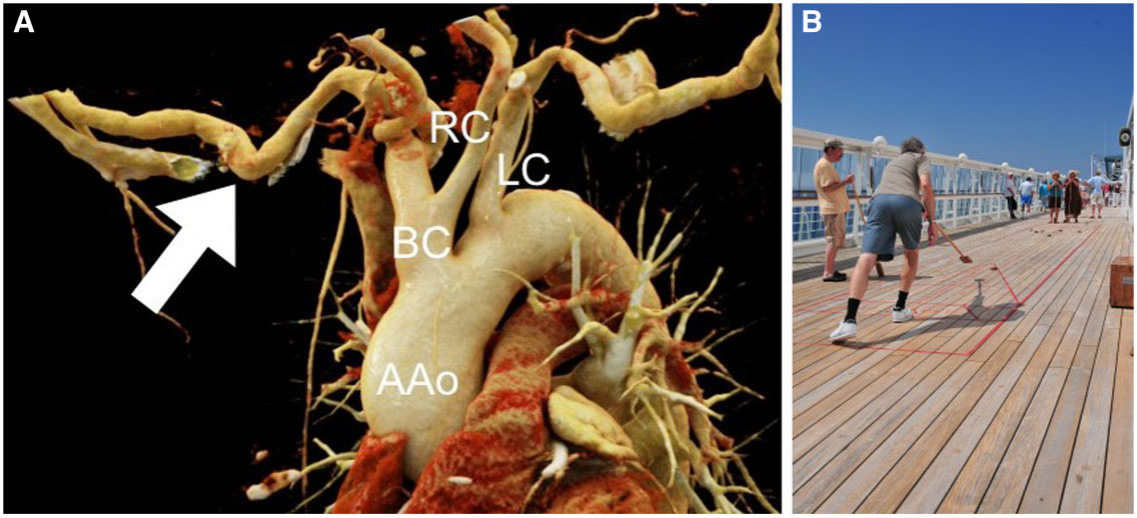
(**A**) 3D volume rendering of computed tomography. Ascending aorta, brachiocephalic artery, left common carotid artery, right common carotid artery, rectangular kinked axillary artery (arrow). (**B**) Shuffleboard on a cruise ship.

The patient developed an unexpected and acute episode of heart failure on the 11th postoperative day. Emergent coronary angiography excluded stenosis or kinking of coronary arteries. Transthoracic echocardiography showed normal valve function but revealed the new onset of a Takotsubo cardiomyopathy. Left ventricular ejection fraction was severely reduced (33%) with the typical pattern of apical ballooning (Video 1). Implantation of a microaxial pump to decompress the left ventricle (Impella 5.5) was scheduled. Preoperative computed tomography showed rectangular kinking of the right subclavian artery (Fig. 1 and Video 2). After intubation and induction of general anaesthesia, a 10-mm Hemashield vascular graft (Getinge AB, Sweden) was sutured to the right axillary artery, tunnelled outside the wound, and a 0.035″ J-Tip guidewire (Amplatz Super Stiff™, Boston Scientific, USA) was passed through the anastomosis into the left ventricle under echocardiographic and fluoroscopic guidance to stretch the kinking of the subclavian artery. The Impella 5.5 was then passed over the guidewire through the anastomosis; however, due to the softness of the Impella 5.5 catheter, it was not possible to push it past the kinking. To supply power directly to the pump we used a 12.0-Fr introducer with a length of 45 cm (Check-Flo Performer^®^ Introducer, Cook Inc., USA) fitted over the guidewire behind the Impella device as a ‘cue stick’ to move the rigid part of the pump forward to overcome the kinking using a ‘shuffleboard technique’ (Fig. 1). Once the pump had slowly reached the ascending aorta, the introducer was removed and the pump placed into the left ventricle. A correct position of the Impella pump was confirmed by transoesophageal echocardiography (Video 1).

The haemodynamic situation stabilized immediately after implantation. The Impella 5.5 was successfully weaned after 6 days of support. The further postoperative course was uneventful and the patient was discharged home on the 20th postoperative day.

## DISCUSSION

**Figure 2:**
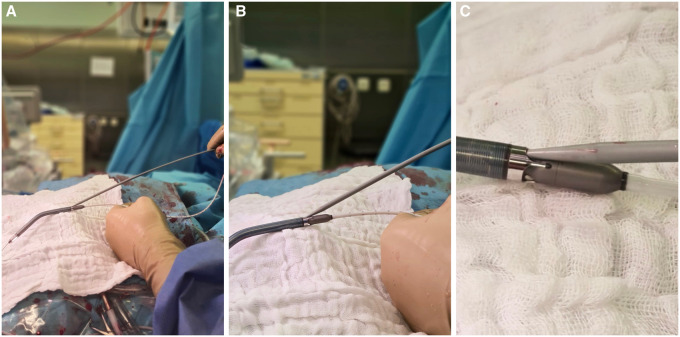
(**A**–**C**) Point of connection between the tip of the dilator and the pump housing.

In most cases, the implantation of microaxial flow pumps is a straightforward procedure [2]. Even though the rigid part of the Impella 5.5 is short (27 mm), it may still not be possible to pass it through a kinked subclavian artery. Therefore, advancing the Impella 5.5 using the ‘shuffleboard technique’ may help overcome this obstacle. The technique may be used only if stenosis or occlusion of the artery is ruled out by a computed tomography scan or intraoperative angiography. The risk of dissection or perforation of the artery should be considered. In this situation, removal of the Impella 5.5 and stent implantation would be indicated. Furthermore, the stylet of the introducer may damage the pump and consequently cause malfunction. We verified the compatibility of the tip of the 12-Fr dilatator with the housing of the pump (Fig. 2). Therefore, implantation of the Impella CP in combination with veno-arterial ECLS through the prosthesis (ECMELLA 2.0 procedure) [3] or the femoral artery (ECMELLA 1.0 procedure) [4] may be a less risky alternative. The advantages of the Impella 5.5 are early mobilization of the patient and full haemodynamic support. In our case, the patient required 5.5 l/min blood flow, while inotropic support—which is not advised in Takotsubo CMP—could be completely weaned.

## CONCLUSION

In the event of (rectangular) kinking of the subclavian artery, the ‘shuffleboard technique’ employing a long introducer may facilitate successful positioning of the microaxial left ventricular assist device (LVAD).

**Conflict of interest:** LW: None. JK: Grants or contracts from any entity: Edwards, LivaNoval. Payment or honoraria for lectures, presentations, speakers bureaus, manuscript writing or educational events: Edwards, Medtronic, Abbott, LivaNova, Cryolife. Leadership or fiduciary role in other board, society, committee or advocacy group, paid or unpaid: TC EACTS, ECSC Board, ISMICS Board. VF: Grants or contracts from any entity: Medtronic GmbH, Biotronik SE & Co., Abbott GmbH & Co. KG, Boston Scientific, Edwards Lifesciences, Berlin Heart, Novartis Pharma GmbH, JOTEC/Cryolife GmbH, LivaNova, Zurich Heart (I hereby declare that I have relevant (institutional) financial activities outside the submitted work with the mentioned commercial entities in relation to Educational Grants (including travel support), fees for lectures and speeches, fees for professional consultation, research and study funds. EVP: Consulting fees: Abbott (institutional grants), Medtronic (institutional grants), Abiomed (institutional grants). Payment or honoraria for lectures, presentations, speakers bureaus, manuscript writing or educational events: Abbott (institutional grants), Medtronic (institutional grants), Abiomed (institutional grants). Support for attending meetings and/or travel: Abbott (institutional grants), Medtronic (institutional grants), Abiomed (institutional grants). Participation on a Data Safety Monitoring Board or Advisory Board: Abbott, Medtronic.

## Reviewer information

Interdisciplinary CardioVascular and Thoracic Surgery thanks Roman Gottardi, Dominik Wiedemann and the other, anonymous reviewer(s) for their contribution to the peer review process of this article.

## Author contributions

**Leonhard Wert**: Conceptualization; Visualization; Writing—original draft; Writing—review & editing. **Jörg Kempfert**: Writing—review & editing. **Volkmar Falka**: Writing—review & editing. **Evgenij V. Potapova**: Conceptualization; Writing—review & editing.
